# Usefulness of Sarilumab in Patients with Rheumatoid Arthritis after Regression of Lymphoproliferative Disorders

**DOI:** 10.1155/2023/5780733

**Published:** 2023-11-06

**Authors:** Yoshifumi Tada, Akira Maeyama, Tomonobu Hagio, Mariko Sakai, Akihito Maruyama, Takuaki Yamamoto

**Affiliations:** ^1^Department of Rheumatology, Faculty of Medicine, Saga University, Saga, Japan; ^2^Department of Orthopaedic Surgery, Faculty of Medicine, Fukuoka University, Fukuoka, Japan

## Abstract

Lymphoproliferative disorders (LPDs) are serious complications associated with rheumatoid arthritis (RA) treatment that mostly occur during methotrexate (MTX) treatment. Cessation of MTX may induce regression of LPDs but is often followed by a flare of RA. Here, we describe two patients with RA flares after the discontinuation of MTX due to LPDs and sarilumab was useful for the treatment of RA without a relapse of LPDs. Patient 1 was an 84-year-old woman, who developed an LPD in the pharyngeal region after 7 years of MTX treatment. Discontinuation of MTX induced regression of LPD but RA flared within 6 months. Administration of sarilumab, in addition to salazosulfapyridine and prednisolone, reduced the RA activity without LPD relapse. Patient 2 was a 76-year-old man, who developed LPD in the pharyngeal region after 5 years of MTX treatment. Discontinuation of MTX induced regression of LPD, but soon RA flared. Although treatment with tocilizumab (TCZ) was effective in controlling RA, it flared again after 2 years. TCZ was switched to sarilumab and RA was in remission. LPD did not recur during these periods.

## 1. Introduction

Lymphoproliferative disorders (LPDs) are serious complications associated with rheumatoid arthritis (RA) treatment that mostly occur during treatment with methotrexate (MTX) [1, 2]. It was recently classified as another iatrogenic immunodeficiency-associated LPD in the revised fourth edition of the World Health Organization classification [3]. RA-related LPDs are mainly seen in senile patients and are characterized by frequent extranodal lesions in the lung, oral cavity, pharynx, and stomach; diffuse large B-cell lymphoma (DLBCL); and the presence of Epstein–Barr virus (EBV) [1, 4]. In addition, discontinuation of MTX sometimes induces a regression of LPDs [5, 6]. The 5-year survival rate for LPDs was reported to be 59–78% in Japan [7–9]. In these patients, it is challenging to treat RA without LPD relapse. Rituximab is recommended in these cases but is not approved for RA in Japan and cannot be used for CD20-positive LPDs once a complete response is achieved [10]. Tocilizumab (TCZ) has shown the highest retention rate among biological disease-modifying antirheumatic drugs (bDMARDs) after regression of LPDs in patients with RA, suggesting the usefulness of the anti-interleukin (IL)-6 agents in these patients [6]. Here, we describe two patients with RA who flared after the discontinuation of MTX due to LPDs, and sarilumab was useful for the treatment of RA without a relapse of LPDs.

## 2. Case Reports

### 2.1. Case 1

**Figure 1:**
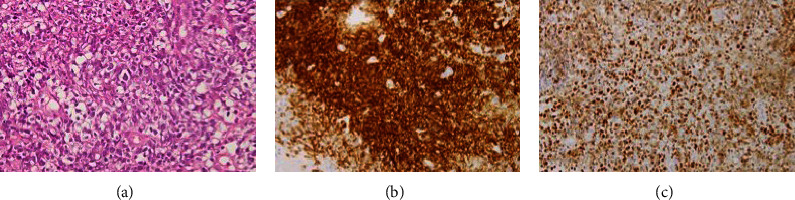
Lymphoproliferative disorders in patient 1. (a) Pathology of the mass shows medium-to-large lymphoid cells with pale cytoplasm and polymorphic nuclei (H&E stain ×200). (b) Immunohistochemical staining shows that the lymphoid cells are positive for CD20. (c) Lymphoid cells are positive for EBV-encoded small RNA in *in situ* hybridization. H&E: hematoxylin and eosin; EBV: Epstein–Barr virus.

An 84-year-old woman who had been treated for RA with MTX (6–8 mg/w) for 6 years was referred to our hospital after developing a mass at the base of the tongue. Chest computed tomography revealed hilar and mediastinal lymph node swellings. RA activity was in remission (disease activity score (DAS) 28-CRP: 1.32) and laboratory investigations revealed white blood cells (WBCs) of 4600/mm^3^, lactate dehydrogenase (LDH) of 326 U/L, C-reactive protein (CRP) of 0.17 mg/dL, soluble IL-2 receptor (sIL-2R) of 1705 U/mL (reference 122–496 U/mL), RF of 35 IU/mL, and anti-cyclic citrullinated peptide antibody (ACPA) of 67.4 U/mL. Biopsy of the mass showed medium-to-large lymphoid cells with pale cytoplasm and polymorphic nuclei (Figure 1(a)). Immunohistochemical staining showed that lymphoid cells were positive for CD20 and EBV-encoded small RNA was positive in *in situ* hybridization (Figures 1(b) and 1(c)). The patient was diagnosed with an EBV-positive DLBCL on the basis of systemic lymphadenopathy and aggressive B-cell LPD. The mass and lymph nodes regressed after discontinuation of MTX; however, 6 months later, the RA flared. Treatment with salazosulfapyridine and prednisolone (PSL) at 5 mg/day was ineffective and the RA activity remained very high (DAS28-CRP: 6.3, Clinical Disease Activity Index (CDAI): 34.5, and CRP: 6.6 mg/dL). We commenced sarilumab 300 mg subcutaneously every other week to successfully reduce the RA activity. CDAI decreased to 12.6, 10.6, and 4.1 at 16, 24, and 52 weeks, respectively. The patient had been treated with sarilumab for 5 years. At the last visit, the CDAI was 1.0 and the LPD lesions disappeared.

### 2.2. Case 2

**Figure 2:**
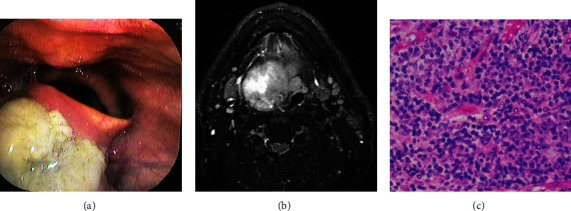
Lymphoproliferative disorders in patient 2. (a) Laryngoscopy shows a mass in the midpharynx. (b) Gadolinium-enhanced magnetic resonance imaging shows a mass (47 × 37 × 52 mm) in the midpharynx. (c) Histopathology of the mass shows an accumulation of atypical lymphoid cells (H&E stain ×200). A diagnosis of DLBCL is made. H&E: hematoxylin and eosin; DLBCL: diffuse large B-cell lymphoma.

A 76-year-old man was diagnosed with RA with positive ACPA and RF results and had been treated with MTX for 5 years. The patient had throat pain and visited an otorhinolaryngologist; a pharyngeal mass was noted for which he was referred to our hospital. Laryngoscopy (Figure 2(a)) and gadolinium-enhanced magnetic resonance imaging revealed a mass (47 × 37 × 52 mm) in the midpharynx (Figure 2(b)). However, the lymph nodes remained unswollen. Laboratory investigations showed WBCs of 6400/mm^3^, LDH of 163 U/L, CRP of 0.69 mg/dL, and sIL-2R of 1091 U/mL. A biopsy of the mass was performed and the histopathology showed an accumulation of atypical lymphoid cells that were positive for CD20, CD79a, and Bcl-6, whereas negative for CD3 and CD30 (Figure 2(c)). No EBV infection was detected. The pathological diagnosis was DLBCL. The pharyngeal mass regressed 3 weeks after discontinuing MTX but the RA flared at the same time (CDAI: 33.7 and CRP 6.98: mg/dL). Administration of TCZ rapidly reduced the RA activity and CDAI decreased to 1.30 by 6 months. However, RA relapsed 2 years later. The patient presented with pain and swelling of the right shoulder, several proximal interphalangeal joints, and both knees with spontaneously ruptured Baker's cysts. Laboratory investigations showed WBCs of 7500/mm^3^, CRP of 0.11 mg/dL, erythrocyte sedimentation rate of 29 mm/h, RF of 1373 IU/mL, ACPA of 244 U/mL, and sIL-2R of 605 U/mL. RA activity was moderate (CDAI: 18.7) and the LPDs did not relapse at that time. TCZ was terminated and sarilumab 300 mg was subcutaneously administered every other week in addition to PSL 5 mg/day. RA activity gradually decreased to a remission; CDAI was 11.0, 7.7, 2.5, and 4.1 at 6, 12, 24, and 36 months, respectively, and PSL was tapered to 3 mg/day. No LPD recurrence was observed during this period.

## 3. Discussion

Here, we described two patients with RA who developed LPDs and RA flares after discontinuing MTX, which was controlled by treatment with sarilumab. To the best of our knowledge, the beneficial role of sarilumab in this context has not been previously reported. Discontinuation of MTX in patients with LPD sometimes leads to an increased RA disease activity and requires treatment intensification [11, 12]. In particular, high disease activity requiring strong treatment, including bDMARDs, poses a challenge in clinical practice. Nakano et al. reported that the 1-year continuation rate was 59% in 38 patients who initiated bDMARDs after regression of LPD, which was lower than that in general patients with RA, and the main reason for discontinuation was the lack of efficacy [12]. A recent multicenter retrospective study in Japan (LPD-WG study) showed that in 88 patients with RA whose LPD had regressed, the continuation rate of bDMARDs was 67.8% and the risk factors for discontinuation of bDMARDs were persistent LPD, non-DLBCL, and a high clinical disease activity of RA [6]. TCZ showed the highest retention rate among bDMARDs, particularly in DLBCL. Tumor necrosis factor (TNF) inhibitors and abatacept showed a greater lack of efficacy than TCZ and TNF inhibitors also caused more adverse events [6].

In our study, both patients were successfully treated with sarilumab, an anti-IL-6 receptor antibody. Sarilumab was used as the first bDMARD against RA flare after discontinuing MTX in case 1 and as a second bDMARDs after a secondary lack of efficacy of TCZ in case 2. In both patients, sarilumab showed remarkable effects on RA and induced remission without LPD relapse. In case 2, TCZ suddenly lost efficacy after 2 years of treatment, suggesting antidrug antibody production; however, this may have been a very rare occurrence, since the antibody production rate against TCZ is very low [13]. Sarilumab treatment has been shown to be effective in patients with an inadequate response to TCZ [14].

Relapse and survival of patients with LPDs have been reported to depend on their pathological type. Event-free survival was better in polymorphic-type LPDs than in DLBCL, classic Hodgkin's lymphoma, and peripheral T-cell lymphoma [15], and progression-free survival was the greatest for reactive lymphoid hyperplasia, followed by polymorphic-LPDs, DLBCL, and classic Hodgkin's lymphoma [9]. In addition, classic Hodgkin's lymphoma has been reported to be a risk factor for LPD relapse [6].

DLBCL is the most common type of LPD in Japan, accounting for 50–60% of biopsied cases [2]. Recently, a close relationship between DLBCL and IL-6 was reported. Hashwah et al. showed that IL-6 activates and induces the proliferation of a subset of DLBCL cells that express IL-6 receptors, indicating that the IL-6 pathway is vital in some DLBCL cases and TCZ reduces the growth of primary DLBCL cells or DLBCL cell lines in various therapeutic settings [16]. This study suggests that IL-6 inhibition suppresses DLBCL expansion and mitigates synovitis in patients with RA. These data and our experiences suggest that sarilumab may be as useful as TCZ, since both share a common mode of action, in patients with RA-associated regression of LPDs (particularly DLBCLs).

Activation of the EBV has been shown in a population with LPDs. A systematic review reported that the prevalence of EBV infection in RA-LPD is 54%, with the highest prevalence found in Asia (65%), followed by North America (39%) and Europe (22%) [4]. Moreover, there is a significant association between EBV infection and LPD susceptibility in patients with RA [4]. In our patients, case 1 was positive and case 2 was negative for EBV. Regarding EBV infection and DMARDs, increased levels of EBV have been observed in patients with RA or polymyositis who were treated with regimens including MTX compared to those without MTX [17]. These results suggest that MTX promotes the reactivation of latent EBV. In contrast, one study reported that long-term treatment with abatacept (34% associated with MTX) or TCZ (37% associated with MTX) did not increase the EBV load in the peripheral blood mononuclear cells of patients with RA [18].

Another advantage of anti-IL-6 agents against RA is their usefulness as a monotherapy. Clinical trials have shown that TCZ and sarilumab exert comparable efficacies with and without MTX against RA [19, 20]. This may be an advantage of anti-IL-6 agents, such as in patients with LPD in whom resumption of MTX is difficult.

The American College of Rheumatology recommendation guidelines for the treatment of RA state that rituximab is conditionally recommended over other bDMARDs for patients with a history of previous LPDs for which rituximab is an approved treatment and who have moderate-to-high disease activity [10]. In Japan, long-term rituximab treatment for RA is not permitted. Therefore, the long-term effects of anti-IL-6 therapy should be evaluated from the viewpoint of relapse prevention in patients with RA, especially in those complicated by DLBCL. Our case reports suggest that sarilumab may be useful for patients with RA in these settings. However, additional case reports are needed to validate our findings.

## Data Availability

The laboratory and imaging data used to support the findings of this study are available from the corresponding author upon request.
