# Correction to “Usefulness of Sarilumab in Patients with Rheumatoid Arthritis after Regression of Lymphoproliferative Disorders”

**DOI:** 10.1155/crrh/9864275

**Published:** 2025-10-03

Y. Tada, A. Maeyama, T. Hagio, M. Sakai, A. Maruyama, and T. Yamamoto, “Usefulness of Sarilumab in Patients with Rheumatoid Arthritis after Regression of Lymphoproliferative Disorders,” *Case Reports in Rheumatology* 2023 (2023): 5780733, https://doi.org/10.1155/2023/5780733.

In the article titled “Usefulness of Sarilumab in Patients with Rheumatoid Arthritis after Regression of Lymphoproliferative Disorders,” there were errors in units in the sections 2.1 and 2.2. These errors are shown below:

“We commenced sarilumab 300 mg subcutaneously every other week to successfully reduce the RA activity” should read “We commenced sarilumab 200 mg subcutaneously every other week to successfully reduce the RA activity.”

“RA activity was moderate (CDAI: 18.7) and the LPDs did not relapse at that time. TCZ was terminated and sarilumab 300 mg was subcutaneously administered every other week in addition to PSL 5 mg/day” should read “RA activity was moderate (CDAI: 18.7) and the LPDs did not relapse at that time. TCZ was terminated and sarilumab 200 mg was subcutaneously administered every other week in addition to PSL 5 mg/day.”

We apologize for these errors.

